# mRNA-based platform for preventing and treating *Staphylococcus aureus* by targeted staphylococcal enterotoxin B

**DOI:** 10.3389/fimmu.2024.1490044

**Published:** 2024-11-21

**Authors:** Fumei Luo, Chuanfei Xu, Chengwen Zhang, Aomo Tan, Dongshui Lu, Ping Luo, Ping Cheng, Weijun Zhang, Lijuan Bai, Cuiyun Yu, Si Sun, Hao Zeng, Quanming Zou

**Affiliations:** ^1^ School of Pharmacy, University of South China, Hunan, China; ^2^ National Engineering Research Center of Immunological Products, Third Military Medical University, Chongqing, China; ^3^ Medical Research Institute, Southwest University, Chongqing, China; ^4^ School of Pharmacy and Bioengineering, Chongqing University of Technology, Chongqing, China; ^5^ College of Pharmacy, Chongqing Medical University, Chongqing, China

**Keywords:** *Staphylococcus aureus*, staphylococcal enterotoxin B, mRNA, vaccine, antibody, bacterial infection

## Abstract

*Staphylococcus aureus* (*S. aureus*) possesses numerous virulence factors, with the increasing prevalence of drug-resistant strains heightening the threat posed by this pathogen. Staphylococcal enterotoxin B (SEB), a highly conserved toxin secreted by *S. aureus*, is also recognized as a potential bioweapon with super-antigenic activity. SEB represents a promising target in efforts to combat infections caused by *S. aureus*. We developed mRNA-based vaccine and antibody targeting SEB for both prophylactic and therapeutic purposes in varying *S. aureus* infection conditions. The mSEB mRNA vaccine (10 μg per mouse) induces more robust and persistent immune responses, including higher antibody titers and specific cellular immune responses, compared to immunization with 30 μg of mSEB protein adjuvanted with aluminum phosphate. Additionally, the anti-SEB mRNA antibody maintains secretion of anti-SEB monoclonal antibody (mAb) with a dosage that is 10 times lower than purified protein administration. The mRNA-based antibody exhibits superior pharmacokinetic profiles compared to its protein counterparts, efficiently neutralizing SEB and clearing *S. aureus* from circulation. Both the mRNA vaccine and mRNA antibody demonstrate preventive and therapeutic effects by eliciting specific immune responses and generating high-affinity antibodies in mice. We have laid the groundwork for the development and evaluation of mRNA-based vaccines and antibodies targeting SEB produced by *S. aureus*. Our studies demonstrate that these approaches are more effective than traditional protein-based vaccines and antibodies in terms of inducing immune responses, pharmacokinetics, and their prophylactic or therapeutic efficacy against *S. aureus* infections.

## Introduction

*Staphylococcus aureus* (*S. aureus*) is a significant cause of both community and hospital-acquired infections, largely due to the rise of highly virulent and multi-antibiotic-resistant strains (1). Approximately 20–30% of the population is colonized by *S. aureus*, serving as that increases the risk of subsequent infections or transmission to others (2–4). The prevalence of methicillin-resistant *S. aureus* further exacerbates the threat posed by this pathogen (5). Consequently, there is an urgent need for more effective therapies. Given the limited efficacy of current antibiotics and the high virulence and pathogenicity associated with *S. aureus*, developing safe and effective countermeasures against this bacterium remains a top priority. While *S. aureus* infections typically start as skin infections, they can escalate to life-threatening conditions such as pneumonia, sepsis, and meningitis (6). When *S. aureus* enters the bloodstream causing bacteremia and sepsis, mortality rates as high as 30% have been documented, making it one of the most common serious infections globally (7). Once inside the body, *S. aureus* evades innate defenses by expressing virulence factors, including Staphylococcal enterotoxin B (SEB), a highly conserved toxin that *S. aureus* secretes and which is also considered a potential bioweapon (5, 8, 9). SEB acts as a potent super-antigenic toxin by directly interacting with the major histocompatibility complex class II (MHC II) and specific Vβ regions of the T-cell receptor (TCR). This interaction leads to excessive activation of monocytes/macrophages and T lymphocytes, causing these host cells to produce large amounts of pro-inflammatory cytokines and chemokines. Consequently, this cascade triggers inflammation and coagulation reactions, potentially resulting in severe clinical symptoms. Moreover, SEB is prevalent in many isolates of the predominant Asian community-associated *S. aureus* lineage sequence type (such as ST59 strain), exacerbating the severity of *S. aureus* infections (10). Recognized as the primary pathogenic factor of *S. aureus* and classified as a B-class biological warfare agent by the Centers for Disease Control and Prevention in the United States, SEB is a critical target for developing anti-toxin neutralizing antibodies aimed at preventing and treating *S. aureus* infections (11).

Vaccines and monoclonal antibodies (mAbs) have become essential tools for preventing and treating various diseases, including infections and cancer, and offer a promising approach to combat the rising threat of antimicrobial resistance (AMR) driven by broad-spectrum antibiotics (12–17). Vaccines targeting specific antigens stimulate the production of antibodies through active immunity, providing preventive benefits. Similarly, mAbs that target bacterial toxins represent a promising strategy for treating bacterial infections. For instance, bezlotoxumab, a humanized monoclonal antibody, is approved for treating *Clostridium difficile* infection by targeting *Clostridium difficile* toxin B (18–20). Therefore, developing vaccines or mAbs targeting SEB holds significant promise for combating *S. aureus* infections.

To date, mRNA technology has shown promising results in preclinical studies for developing vaccines and antibodies against various diseases, including infections and cancer (21–24). mRNA platforms enable rapid manufacturing and flexible design tailored to different targets, allowing for sustained endogenous protein secretion *in vivo*. These capabilities facilitate the swift implementation of personalized vaccine and antibody therapies. Several studies have also utilized mRNA technology in antibacterial research in recent years (25–27).

mRNA vaccines have emerged as a promising vaccine format compared to conventional vaccines due to their rapid manufacturability and adaptable design for diverse targets. They can induce robust cellular immune responses even without adjuvants, unlike subunit protein vaccines (28–30). Antibody based antibacterial therapy is considered to be a potential alternative, however, purified mAbs are prone to rapid clearance and degradation, requiring frequent high-dose administrations (typically in the range of mg/kg). This characteristic also heightens the risk of undesirable side effects, including immune system activation, thereby restricting the clinical utility of mAbs (31). mRNA encoding antibodies secreted antibodies endogenously, thereby potentially increasing their effective concentration and duration of action *in vivo* (32–34).

In a previous study, we purified mutated SEB protein (mSEB), which is a critical component of the recombinant five-antigen *S. aureus* vaccine (rFSAV, NCT02804711 and NCT03966040) (35). mSEB effectively reduces toxin level of wild-type SEB (wSEB) while maintaining SEB’s immunogenicity. Additionally, we identified a variable region sequence and engineered an IgG1-type antibody (anti-SEB mAb) with high-affinity binding to SEB from the peripheral blood samples of volunteers who have enrolled in the clinical trial of rFSAV by high-throughput isolation of immunoglobulin genes from single human B cells.

Considering the potent super-antigenic toxin and pathogenicity of SEB in *S. aureus*, and vary resistance strategies under different infection situations, we developed mRNA-based vaccines and antibodies targeting SEB for both prophylactic and therapeutic purposes in varying *S. aureus* infection conditions. In our current study, we developed a mRNA vaccine encoding mSEB protein (mSEB mRNA vaccine) to provide effective preventive protection against SEB and *S. aureus*, and a mRNA encoding anti-SEB mAb (anti-SEB mRNA antibody) to exhibited excellent neutralizing activity against SEB and therapeutic effects to *S. aureus*. Importantly, these mRNA-based formulations showed superiority over their original protein-based counterparts in terms of efficacy.

The mSEB mRNA vaccine (10 μg per mouse) induces more robust and persistent immune responses, including higher serum antibody titers and specific cellular immune responses, compared to immunization with 30 μg of mSEB protein adjuvanted with aluminum phosphate. The anti-SEB mRNA antibody maintains secretion of anti-SEB mAb with 10 times less dosage than purified antibody administration in protein format. The anti-SEB mRNA antibody exhibited superior pharmacokinetic profiles compared to their protein counterparts, effectively and promptly neutralizing SEB and clearing *S. aureus* from circulation. The mRNA vaccine with preventive effects, as well as the mRNA antibody with therapeutic effects, by eliciting specific immune responses and generating high-affinity antibodies in mice upon administration.

Therefore, targeting SEB proves effective against *S. aureus*. Our study represents a pioneering proof-of-concept, demonstrating that mRNA-based vaccine and antibody targeting SEB have the potential to achieve robust preventive and therapeutic effects against *S. aureus* infections, respectively, which is a significant milestone for the development of alternative strategies against toxin and multidrug resistance bacterial infection.

## Results

### mSEB mRNA vaccine induces persistent humoral immunity and robust cellular immunity

**Figure 1:**
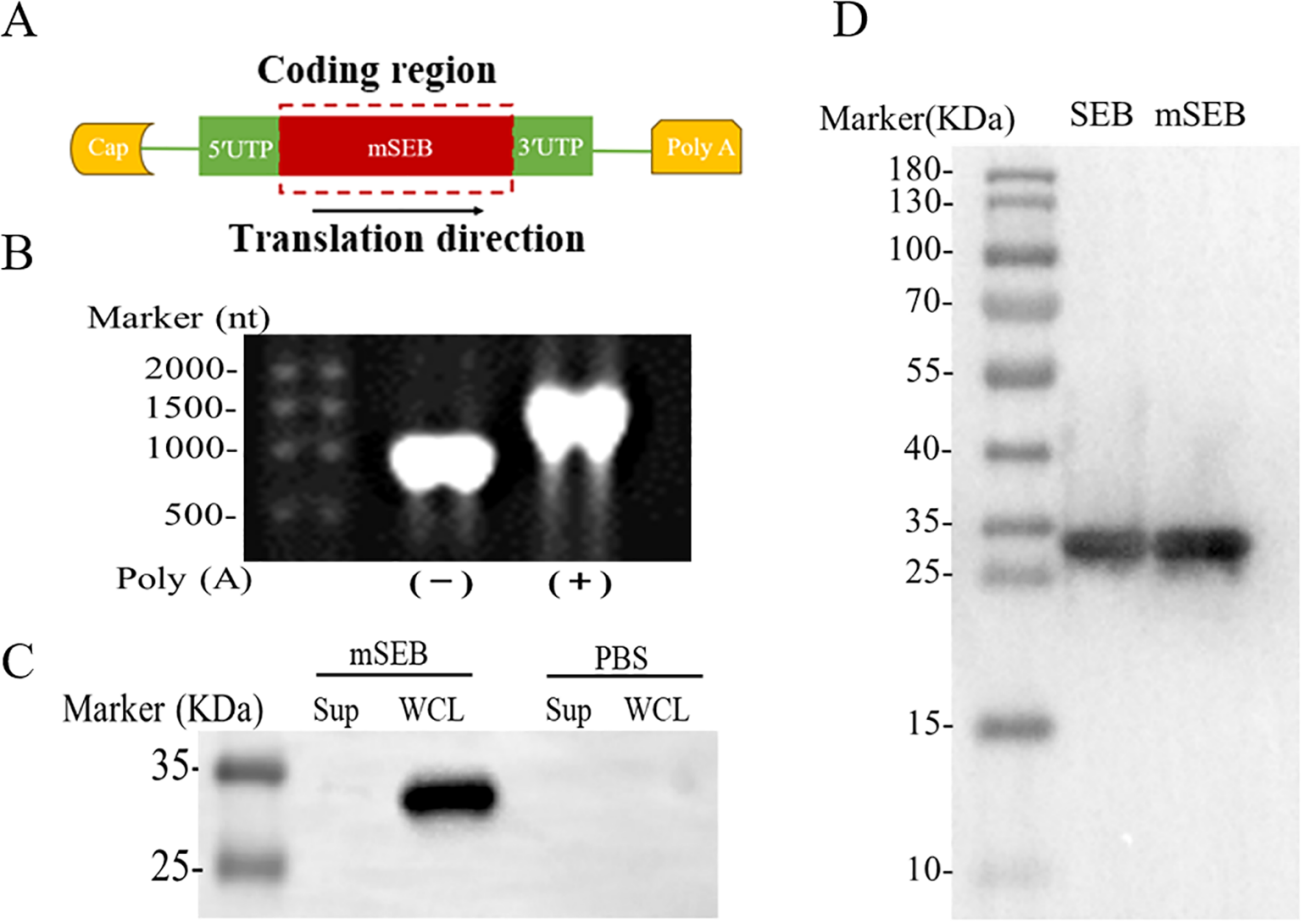
Design and characterization of mSEB mRNA. **(A)** Design of mRNA encoding mSEB. **(B)** Formaldehyde gel electrophoresis analysis of mRNA encoding mSEB. **(C)** The expression of mSEB mRNA 24 hours after transfection into HEK 293T cells detected by WB. It is shown that mRNA were successfully expressed in cells rather than secreted into the supernatants. **(D)** The sera from i.m. injection of mSEB mRNA were used as the primary antibody to detect purified recombinant wild-type SEB (wSEB) or mutant SEB (mSEB) protein by WB.

As shown in **Figure 1A**, the intact mSEB mRNA molecule consists of a 5′ cap, 5′ and 3′ untranslated regions (UTRs), an open reading frame (ORF), and a 3′ poly(A) tail. In our study, the mRNA was designed with a 2′-O-methylated Cap1. In addition, the length of 3′ poly(A)-tail was detected as approximate 110 nt (**Figure 1B**). The expression of mSEB mRNA 48 hours after transfection into HEK 293T cells were detected by western blot (WB). It is shown that mRNAs were successfully expressed in cells rather than secreted into the supernatants (**Figure 1C**). Serum samples collected from mice intramuscularly injected with mSEB mRNA vaccine were used as the primary antibody to detect purified recombinant wild-type SEB (wSEB) or mutant SEB (mSEB) protein via WB. The results indicated the mSEB mRNA vaccine could induce wSEB specific antibodies (**Figure 1D**). Furthermore, we demonstrated the safety of the mSEB mRNA vaccine through CCK-8 cell cytotoxicity assay *in vitro* (**Supplementary Figure 1**). Pathological analysis of tissue samples and evaluation of liver function indices, including alanine aminotransferase (ALT) and aspartate aminotransferase (AST), conducted 24 hours post-administration of the mSEB mRNA vaccine, revealed no significant organ damage or inflammation *in vivo* (**Supplementary Figures 2**, **3**).

**Figure 2:**
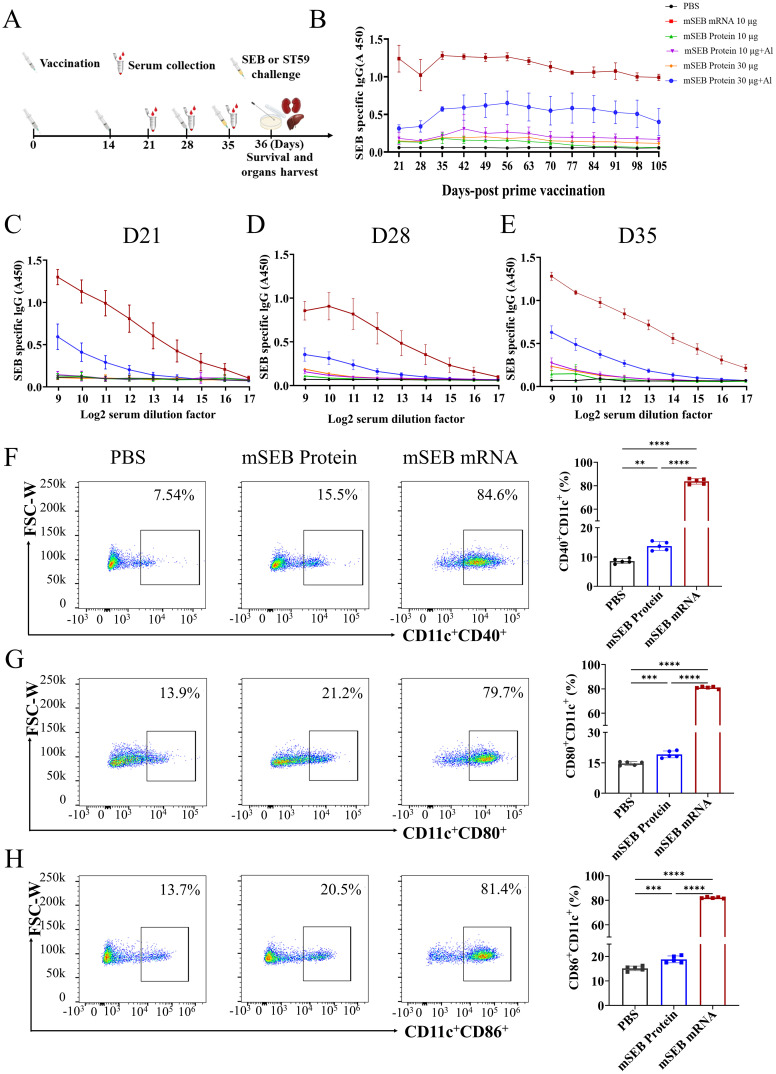
Humoral immune responses after immunization with mSEB mRNA vaccine in mice. **(A)** Schematic diagram of immunization, sample collection and challenge schedule. Mice were immunized on day 0 and boosted with the same dose on day 14 and day 28, respectively. **(B)** OD450 nm values SEB protein-specific IgG in 1:1000 diluted serum samples collected at the indicated time point. **(C)** The SEB-specific IgG antibody titer in in serial diluted serum samples collected at 21 days, **(D)** 28 days and **(E)** 35 days after initial vaccination. **(F)** Expression of CD11c^+^CD40^+^, **(G)** CD11c^+^CD80^+^ or **(H)** CD11c^+^CD86^+^ on BMDCs after 24 hours incubation with mSEB mRNA, mSEB protein and phosphate buffered saline (PBS). Data represent mean ± SD (n= 5 biologically independent samples). One-way ANOVA with Dunnett’s *post-hoc* test was used to determine significance within **(F–H)** (**P< 0.01, ***P< 0.001, ****P< 0.0001).

To determine the immunogenicity of the mRNA vaccine, Balb/c mice were intramuscularly immunized with mSEB mRNA (10 μg per mice), mSEB protein (10 μg per mice with or without alum adjuvant) and mSEB protein (30 μg per mice with or without alum adjuvant). The vaccination schedule followed the timeline depicted in **Figure 2A**, with the prime dose on day 0 and the boost on day 14 and 28, respectively. We confirmed the necessity of the 3nd boost dose by detection of IgG in serum at predetermined time points (**Figure 2B**).

We also confirmed mSEB mRNA vaccine successfully induced higher SEB-specific IgG antibodies in serum. The OD450 nm values of SEB protein-specific IgG were measured in serially diluted serum samples collected at 21, 28, and 35 days after the initial vaccination. Immunization with mSEB mRNA vaccine resulted in significantly higher levels of specific IgG antibodies (including IgG1 and IgG2a subtypes) in serum samples compared to those induced by mSEB protein with or without alum adjuvant (**Figures 2C–E**; **Supplementary Figure 4**). The mSEB protein administration group did not induce obvious IgG antibodies in serum, except for the 30 μg with alum adjuvant. The highest IgG titer within serum from mSEB-mRNA vaccine group reached 1/20000 on day 35 (**Figure 2E**).

We further examined the ability of mSEB mRNA vaccine to promote DC maturation. Compared to mSEB protein group, the mSEB mRNA vaccine induced over three-fold increase in the expression of CD11c^+^CD40^+^, CD11c^+^CD80^+^ or CD11c^+^CD86^+^ on bone marrow derived cells (BMDCs) (**Figures 2F–H**; **Supplementary Figure 5**). Serum samples collected on day 35 from the mSEB mRNA vaccine group displayed significant antibacterial activity, whereas relatively low antibacterial activity was observed in serum from the group receiving 30 μg of mSEB protein with alum adjuvant (**Supplementary Figure 6**).

**Figure 3:**
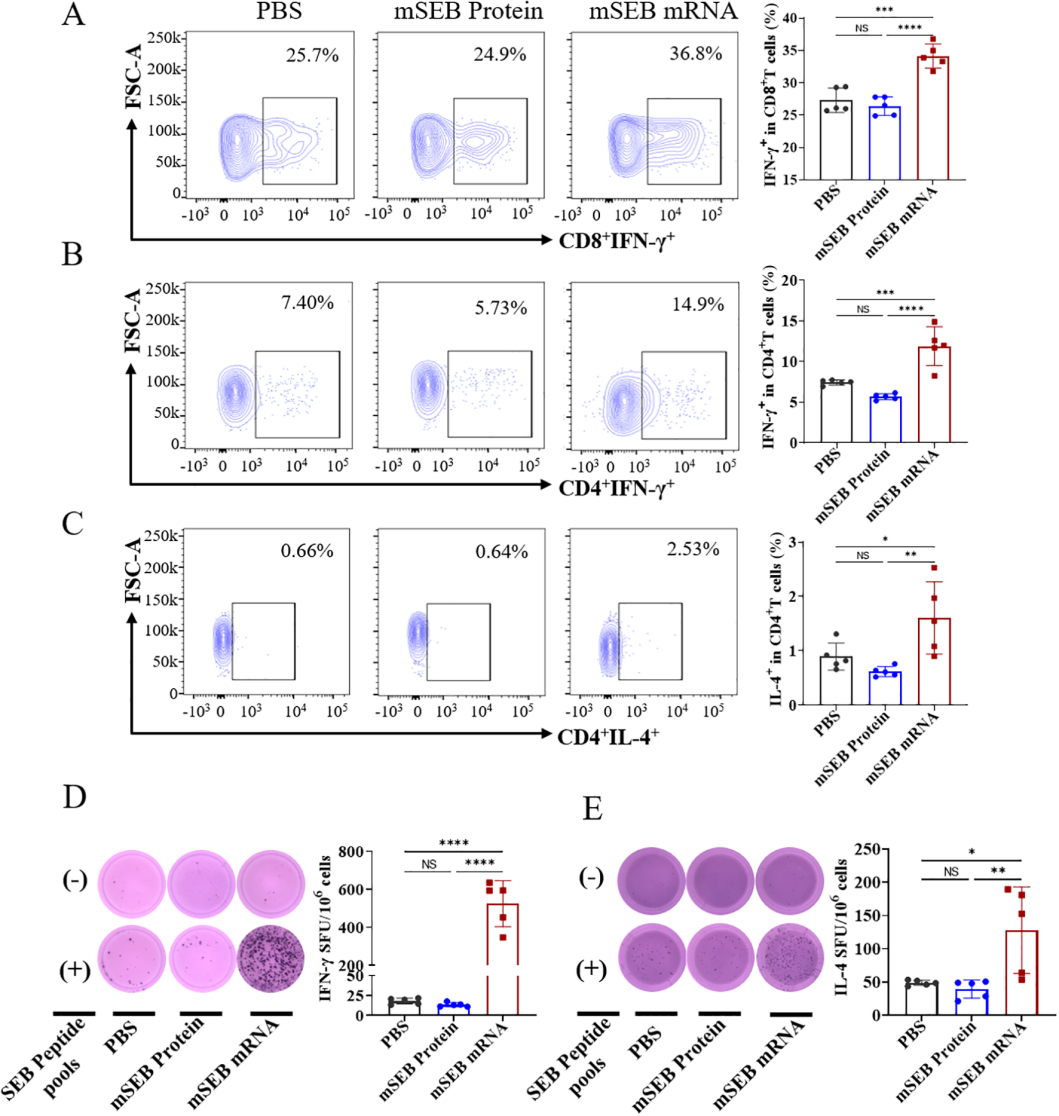
Cellular immune responses induced by mSEB-mRNA vaccinated mice. **(A)** CD8^+^ T cells and **(B)** CD4^+^ T cells in splenic lymphocytes were assayed for IFN-γ^+^ expression by flow cytometry after restimulation with the peptide pools of 15-mer overlapping peptides spanning the SEB protein. **(C)** CD4^+^ T cells in splenic lymphocytes were analyzed for IL-4^+^ expression via flow cytometry in the same way as describe above. **(D)** ELISpot analysis of IFN-γ and **(E)** IL-4 spot-forming cells in splenic lymphocytes after re-stimulation with peptide pools. Data in A-E represent mean ± SD (n = 5 biologically independent samples). One-way ANOVA with Dunnett’s post-hoc test was used to determine significance (NS represents no significant, *P< 0.05, **P < 0.01, ***P < 0.001, ****P < 0.0001).

To investigate cellular immune responses activated by the mSEB mRNA vaccine, we evaluated the intracellular production of interferon-γ (IFN-γ) and interleukin-4 (IL-4) within CD4^+^ and CD8^+^ T cells after ex vivo re-stimulation with 15-mer overlapping peptide pools derived from the SEB protein. Flow cytometry analysis showed that mSEB mRNA vaccine led to significant secretion of IFN-γ^+^ by CD8^+^ (34.0 ± 0.77)% and CD4^+^ T cells (9.85 ± 0.93)% within splenic lymphocytes. However, there was no significant difference in IL-4 secretion by CD4^+^ T cells between mice immunized with mSEB mRNA vaccine and those treated with 30 μg mSEB protein with alum adjuvant (**Figures 3A–C**; **Supplementary Figure 7**). Given the superantigen activity of SEB, the peptide pools derived from SEB protein may non-specifically activate T cells, leading to a robust CD8^+^ T cell responses, including in the PBS group.

We next assessed the production of IFN-γ and IL-4 in splenocytes using Enzyme-linked immunospot analysis (ELISpot). Significantly higher levels of IFN-γ and IL-4 secretion were detected in splenocytes after ex vivo re-stimulation of peptide pools in mSEB mRNA vaccine group, whereas negligible levels of both cytokines were detected in samples from the other groups (**Figures 3D, E**). A similar trend was also observed in the supernatant of splenocyte samples (**Supplementary Figure 8**).

These data demonstrate persistent humoral immune responses and robust cellular immune responses, including specific activation of Th1 cells and cytotoxic T cells, in mice treated with mSEB mRNA vaccine. These findings underscore the significant potential of mSEB mRNA vaccine to achieve potent neutralization of SEB and provide protection against *S. aureus* infections.

### Immunization with mSEB mRNA vaccine efficiently neutralize SEB and prevent *S.aureus* infection in mice

**Figure 4:**
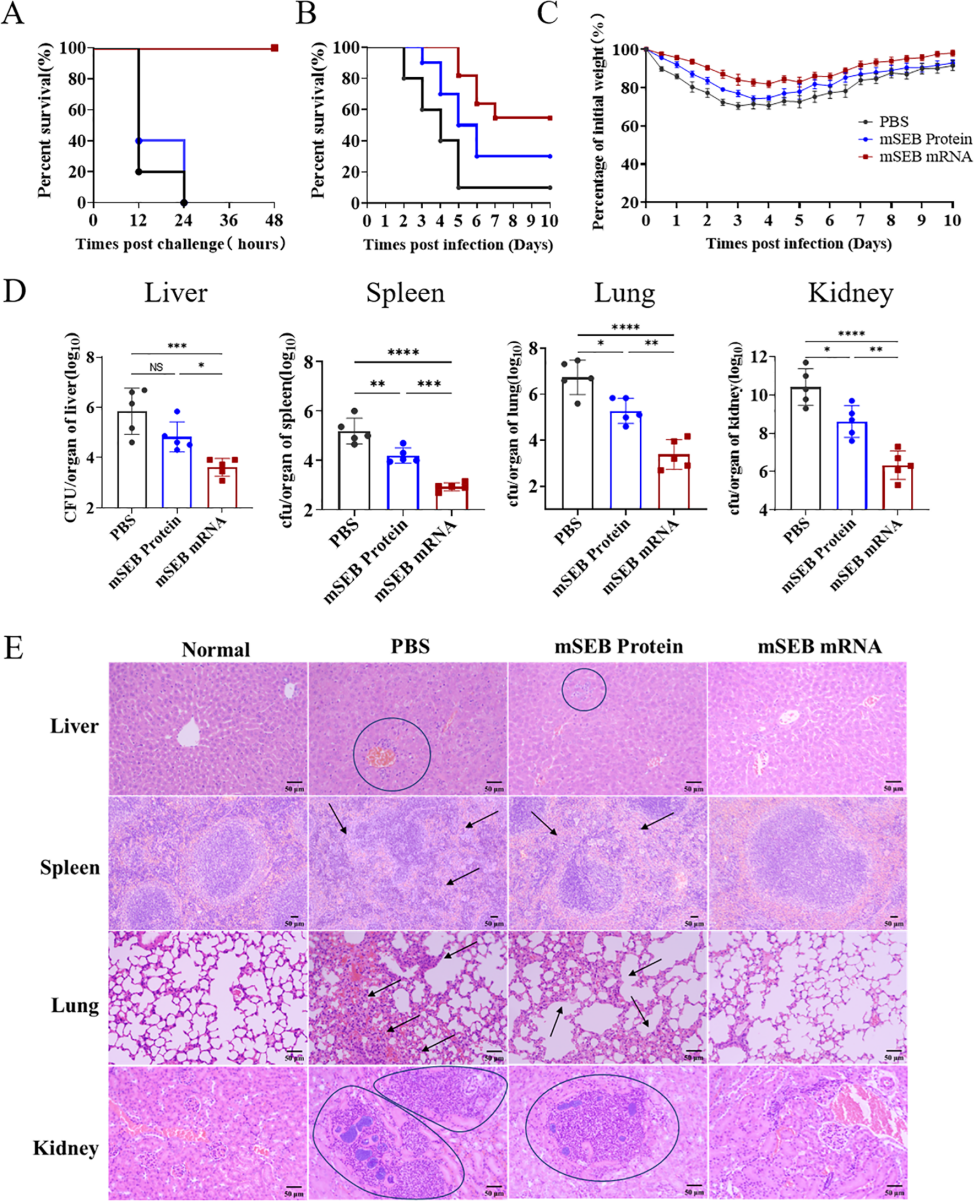
Immunization with mSEB-mRNA vaccine conferred protection against lethal SEB challenge and prevented *S. aureus* infection in mice. The survival rate of mice (n = 10 mice/group) immunized with mRNA vaccine, mSEB protein and PBS after the **(A)** SEB or **(B)** ST59 lethal challenge. **(C)** Animal weights were recorded after challenge. **(D)** CFUs in the organs of mice after challenging. Mice were immunization on day 0, 14 and 28 of 10 μg mRNA vaccine or 30 μg mSEB protein with aluminum adjuvant (PBS as placebo group) and then challenged with ST59 strain at 35 days. The bacterial loads were detected at 24 hour post infection. Data represent mean ± SD (n = 10 mice/group in **(A–C)**, and n = 5 mice/group in **(D)**. One-way ANOVA with Dunnett’s post-hoc test was used to determine significance (NS represents not significant, *P< 0.05, **P < 0.01, ***P < 0.001, ****P < 0.0001). **(E)** Representative hematoxylin-eosin staining (H&E) staining of organs pathology 24 hours after ST59 strain challenge. Scale bars: 50 μm.

The SEB toxin challenge in mice serves as a model for hepatic damage and acute lethality. To assess the *in vivo* neutralizing efficacy of mSEB mRNA, groups of 8-week-old BALB/c mice were challenged post immunization in this established SEB challenge model (**Figure 2A**). The mSEB mRNA vaccine demonstrated remarkable protective efficacy as evidenced by an 100% survival rate, while all mice from control groups were deceased during 48 hours after challenge (**Figure 4A**). The data described above prompted us to confirm if sufficient protection against *S. aureus* could be achieved by immunization of mSEB mRNA vaccine. To this end, a *S. aureus* ST59 strain (a high pathogenic and resistant strain of *S. aureus* in clinical) was applied in the challenge study. In the lethal challenge model of ST59, the mSEB mRNA vaccine demonstrated relatively high protective efficacy of 60% survival rate at the predetermined end (10 days post-challenge), while the protein vaccined group and PBS group were 30% and 10% survival rate, respectively (**Figure 4B**). In the sublethal challenge model of ST59, the body weights of mSEB mRNA vaccinated mice decreased (< 20%) in the first 4 days post-challenge but gradually recovered to a level displayed by the control group (without challenge) (**Figure 4C**). In addition, the mice were euthanized to analysis the bacterial loads in organs (liver, spleen, lung and kidney) 24 hours post-challenge. Significant lower levels of bacterial loads were detected in the organs of mSEB mRNA vaccinated mice compared to control counterparts (**Figure 4D**). Additionally, organ sections from different treatment groups were subjected to histopathological analysis by hematoxylin and eosin (H&E) staining. The organs from healthy mice (normal) and those challenged by ST59 after vaccinating with mSEB mRNA showed normal histological architecture. In contrast, the livers and kidneys of the group administered with PBS (placebo group) or mSEB protein and then challenged by ST59 exhibited significant inflammatory infiltration (**Figure 4E**). Encouragingly, treatment with mSEB mRNA vaccine enhanced the recovery of the damaged liver with no obvious damage or inflammation were observed.

Due to the lack of significant effects in inducing humoral and cellular immune responses, as well as neutralizing SEB toxins in mice, the mSEB protein administration group (30μg per mice with alum adjuvant) exhibited inferior protection effect to mSEB mRNA vaccine group. The results indicated that triple dose immunization of mSEB mRNA vaccine can effectively neutralize SEB in mice by inducing robust SEB-specific immune response, thereby preventing *S. aureus* infection and reducing inflammation, toxicity and hepatic damage.

### Anti-SEB mRNA antibody possess more excellent pharmacokinetic profile to neutralize SEB and exhibit therapeutic efficacy against *S.aureus*

**Figure 5:**
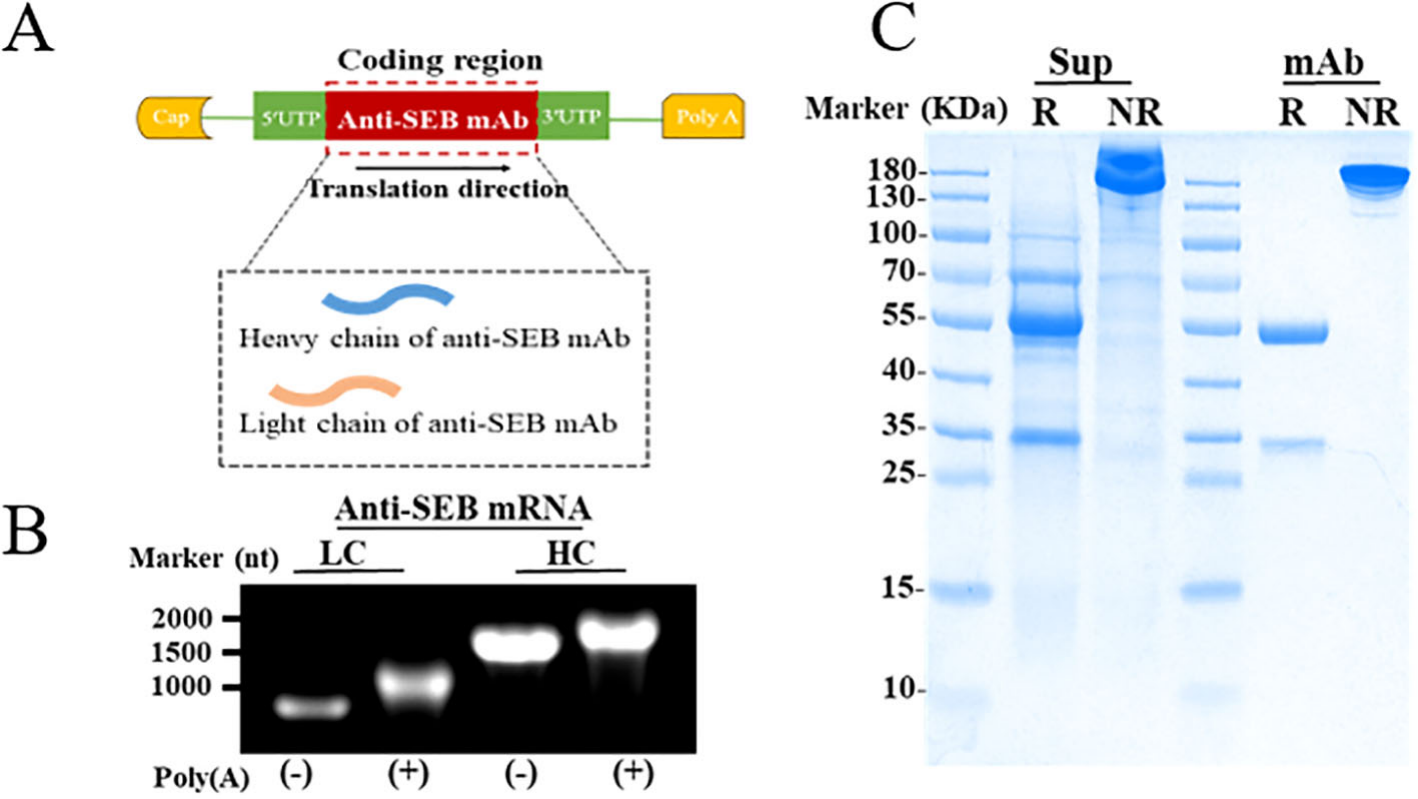
Preparation of anti-SEB mRNA antibody. **(A)** Schematic representation of mRNA constructs for expressing the anti-SEB antibody. **(B)** Formaldehyde gel electrophoresis analysis of mRNA encoding heavy and light chain. **(C)** Reducing (R) and Non-Reducing (NR) SDS-PAGE detection of antibody the in culture supernatants (Sup) from Expi293F after transfection with mRNA antibody; The purified anti-SEB mAb (mAb) was used as the positive control.

As showed in **Figure 5A**, based on sequences of anti-SEB mAb, linear templates incorporating these elements were designed for both the light chain (LC) and heavy chain (HC). Formaldehyde gel electrophoresis confirmed successful preparation of mRNA encoding both LC and HC through *in vitro* transcription, with the 3′ poly(A) tail approximately 110 nucleotides long (**Figure 5B**). Subsequently, lipid nanoparticles (LNPs) were utilized to encapsulate the mRNAs for efficient delivery *in vivo* and *in vitro*. Transfection of the anti-SEB mRNA antibody into multiple cell lines (Raw264.7, Expi293F, and Expi293T) resulted in significant expression of the antibody in the culture supernatant (**Supplementary Figure 9**). SDS-PAGE analysis further demonstrated that the antibody expressed in the supernatant after transfection with Expi293F maintained the same integrity as the purified mAb (**Figure 5C**).

**Figure 6:**
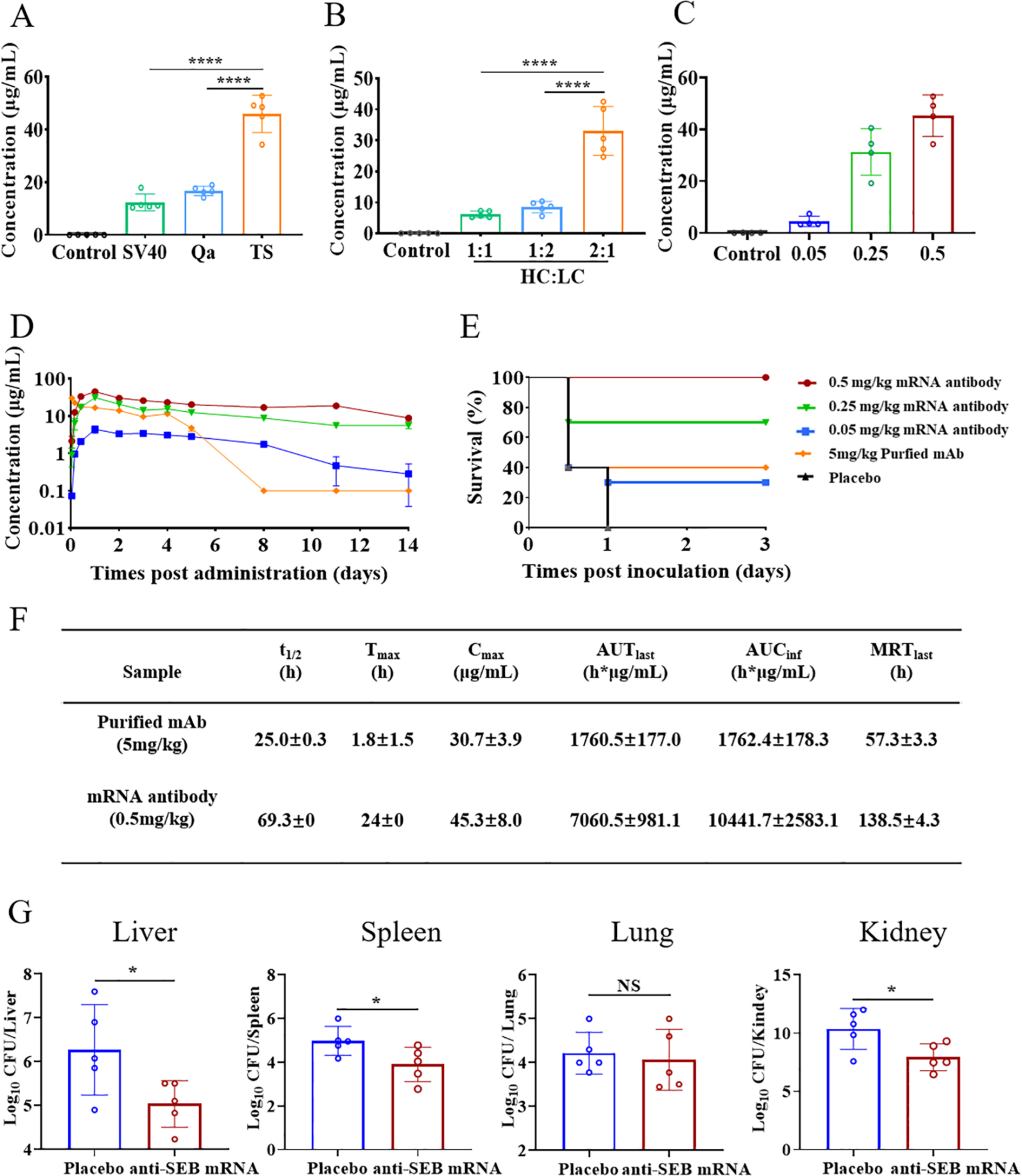
Optimized design, pharmacokinetic profile and neutralizing activity of anti-SEB mRNA antibody. **(A)** Investigation of the impact of different UTRs and **(B)** HC/LC mass ratio on mRNA expression *in vivo* using ELISA assay. **(C)** Serum antibody concentrations at 24 hours post-administration of mRNA antibody. **(D)** Temporal serum antibody concentrations following administration of 0.05, 0.25, and 0.5 mg/kg mRNA antibody and 5 mg/kg purified anti-SEB antibody. **(E)** Survival analysis of mice in the mRNA antibody and Placebo groups 3 days after challenge with SEB. Balb/c mice were challenged with SEB 1 day after a single administration of mRNA antibody or purified antibody. **(F)** Pharmacokinetic analysis of antibodies in serum after administration of a single dose of 0.5 mg/kg mRNA antibody and 5 mg/kg purified anti-SEB antibody. Calculations were performed using DAS software. **(G)** CFUs in the organs of mice in therapeutic administration of mRNA antibody. Mice were infected with ST59 strain and then administration of single-dose 0.5mg/kg mRNA antibody or PBS (placebo group) 1 hour later. The bacterial loads were detected at 24 hours post infection. Each symbol in **(A–C, G)** represents a replicate with mean ± SD depicted. Statistical significance in A and B was determined using one-way ANOVA with Dunnett’s *post-hoc* test (****P < 0.0001). *t-*test was used to determine significance in G, (NS represents not significant, * P < 0.05).

In this study, the influence of different UTR sequences on mRNA expression was investigated, leveraging previous findings. Specifically, UTRs derived from Trastuzumab (TS) were found to significantly enhance antibody secretion *in vivo* following intravenous administration, compared to UTRs from simian virus 40 (SV40) and Exin21/Qa1,2 (Qa) (**Figure 6A**). Furthermore, optimizing the heavy chain to light chain (HC/LC) molar ratio to 2:1 resulted in the highest antibody expression relative to other ratios (**Figure 6B**). Based on these optimization experiments, mRNA constructs incorporating Trastuzumab UTRs and a HC/LC molar ratio of 2:1 were selected for subsequent studies.

Next, we investigated the dose-dependent production of efficient antibodies in vivo using anti-SEB mRNA antibody. Balb/c mice aged 8 weeks received a single intravenous injection of mRNA antibody at doses of 0.05, 0.25, and 0.5 mg/kg (n=4 per group). Serum samples collected 24 hours after injection revealed a dose-dependent increase in antibody concentration of serum (**Figure 6C**). Specifically, the average concentrations of antibody in serum were 4.45 μg/mL, 31.27 μg/mL, and 45.28 μg/mL for the 0.05, 0.25, and 0.5 mg/kg administration groups, respectively.

Subsequently, we monitored the temporal changes in serum antibody concentration (**Figure 6D**). Antibodies from the mRNA administration group were detectable as early as 4 hours post-administration, peaking at 24 hours. Moreover, these mRNA-derived antibodies exhibited sustained expression in a dose-dependent manner, maintaining relatively high concentrations compared to purified antibodies administered at 5 mg/kg.

We further assessed the in vivo pharmacokinetic profile of 0.5 mg/kg anti-SEB mRNA antibody in mice and compared it with 5 mg/kg of purified recombinant anti-SEB antibody (**Figure 6F**). The average serum antibody concentration was detected at 4 hours following administration of 0.5 mg/kg mRNA antibody. It peaked at 45.3 μg/mL at 24 hours and gradually declined to levels below the detection limit over the next 14 days, with an average half-life (t_1/2_) of 69.3 hours. The mean residence time (MRT) was calculated as 138.5 hours. Additionally, the mean AUC_last_ and AUC_inf_ were determined to be 7060.5 and 10441.7 hours × μg/mL, respectively.

In contrast, serum antibody concentration was detectable immediately after injection of 5 mg/kg purified anti-SEB antibody. The peak mean concentration was 30.7 μg/mL, followed by a rapid decline to baseline within 8 days, with an average half-life of 25.0 hours. These results highlight that administration of anti-SEB mRNA antibody can rapidly induce antibody production within 4 hours *in vivo*. Despite the dosage of recombinant anti-SEB antibody (5 mg/kg) being 10 times higher than that of anti-SEB mRNA antibody (0.5 mg/kg), the mRNA-based approach exhibits a more favorable pharmacokinetic profile, achieving higher peak levels compared to the purified antibody group.

As anticipated, all animals that received mRNA encoding the antibody demonstrated dose-dependent protection against SEB challenge. Survival curve analysis showed that mice administered 0.5 mg/kg of mRNA antibody survived throughout the entire observation period, contrasting starkly with the placebo group, which succumbed within 24 hours after challenge. Notably, mice receiving 0.25 mg/kg of mRNA antibody also exhibited significant survival rates, with 70% of the animals surviving, surpassing the survival rate of those treated with 5 mg/kg of purified recombinant mAb (**Figure 6E**).

The anti-SEB mRNA antibody administered at 0.5 mg/kg demonstrated a notable ability to mitigate the cytokine storm induced by SEB, a potent super-antigen that triggers abnormal secretion of cytokines such as TNF-α, IFN-γ, IL-6, and IL-4 (**Supplementary Figure 10**). This effect underscores the potential of anti-SEB mRNA antibody in managing SEB-induced immune responses. Additionally, evaluation of liver function indices, including alanine aminotransferase (ALT) and aspartate aminotransferase (AST), in mice at 12 hours post SEB challenge indicated that the administration of anti-SEB mRNA antibody resulted in relatively mild hepatic damage (**Supplementary Figure 11**).

Next, we aimed to evaluate the *in vivo* therapeutic efficacy of anti-SEB mRNA antibody against *S. aureus* infection using a well-established mouse model. Groups of 8-week-old BALB/c mice received a single dose of 0.5 mg/kg anti-SEB mRNA antibody 1 hour after being challenged with the ST59 strain. As expected, mice treated with PBS (placebo group) exhibited significantly higher bacterial burdens in the liver, spleen, and kidney compared to those receiving anti-SEB mRNA administration. However, no significant difference in bacterial burden was observed in the lungs (**Figure 6G**). These findings underscore that anti-SEB mRNA antibody effectively neutralizes SEB and can therapeutically manage *S. aureus* infection by inducing the secretion of antibodies *in vivo*. This approach reduces inflammation, toxicity, and hepatic damage, demonstrating efficacy in treating bloodstream infections caused by *S. aureus* in mice.

## Discussion

The escalating prevalence of drug-resistant *S. aureus* amplifies the threat posed by this pathogen. Urgent action is required to address the lack of effective antibiotics and the heightened virulence and pathogenicity of *S. aureus*. The development of safe and potent countermeasures against *S. aureus* remains paramount. The widespread emergence of drug resistance has prompted the exploration of bacteria-specific strategies for prophylaxis and therapy, with a focus on mRNA technologies.

In recent years, mRNA-based therapeutic agents have emerged in clinical trials for both prophylactic and therapeutic purposes (36, 37), spanning infectious disease vaccines, gene therapy, and antibody therapy. The flexible design and rapid manufacture of mRNA encoding antigen or antibody have been well demonstrated. Here, we utilized the established mRNA LNP platform, recognized for its efficacy in delivering diverse proteins *in vivo*, as a cornerstone of our study.

**Figure 7:**
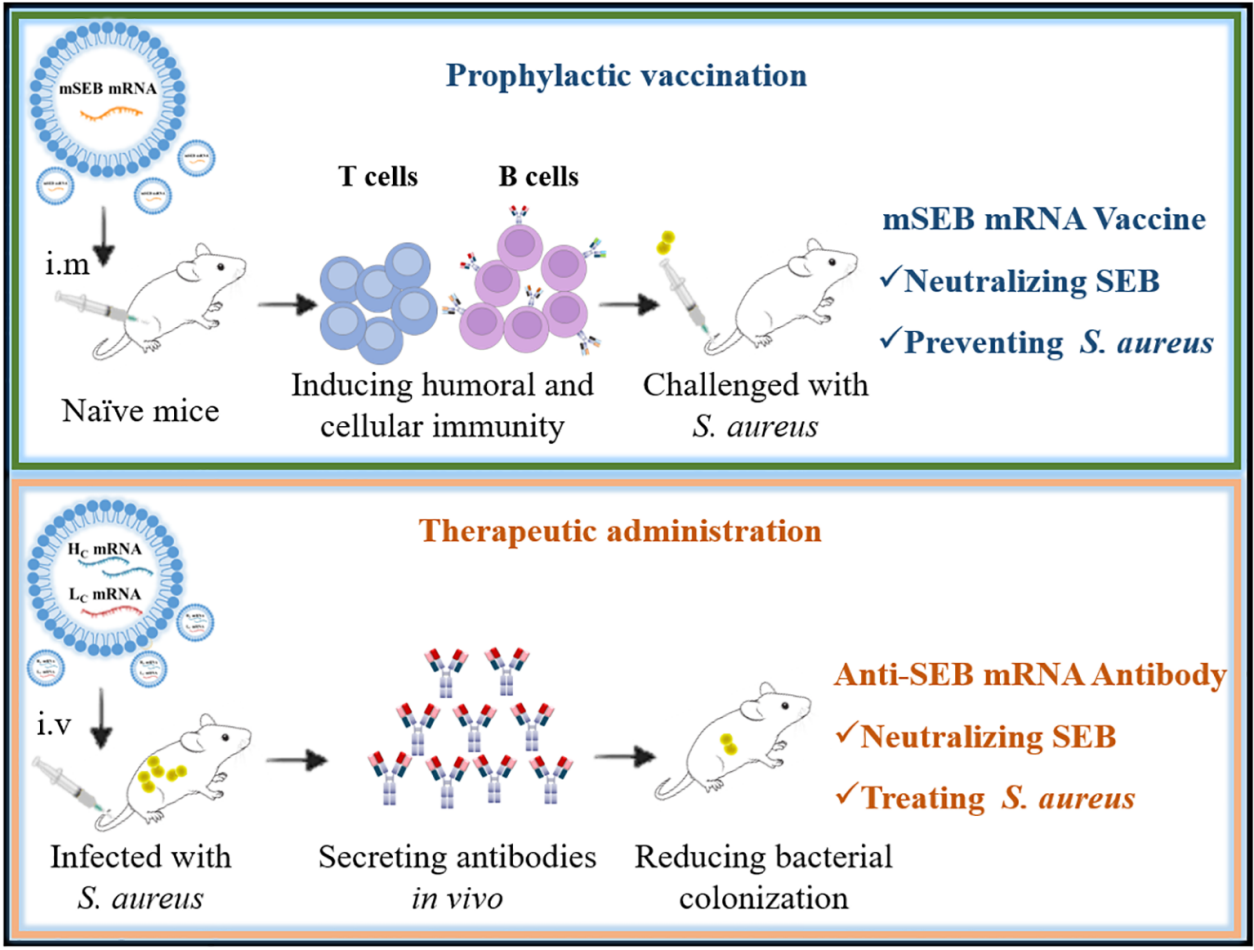
Graphical abstract illustrating mRNA vaccine for prophylactic protection (top) and mRNA antibody for therapeutic effects (bottom).

Effective early vaccination strategies are crucial for reducing the risks of severe infections. Additionally, rapid administration of antibodies is vital for individuals already infected with *S. aureus*. Distinct from tumors or viruses, *S. aureus* possesses numerous virulence factors. SEB, a toxin secreted by *S. aureus* and highly conserved across its species (e.g., ST59 strain), was identified as a critical protective antigen. Based on our previous work, we identified a mutated SEB protein sequence (mSEB) as a crucial protective antigen, additionally, we engineered a humanized monoclonal antibody with high-affinity binding to wild-type SEB (wSEB). Our study represents a pioneering proof-of-concept, demonstrating that mRNA-based vaccines or antibodies targeting SEB have the potential to achieve robust preventive or therapeutic effects against *S. aureus* infections, respectively. This marks a significant milestone in developing alternative strategies against multidrug-resistant bacterial infections (**Figure 7**).

Due to the inherent adjuvant effect of the mRNA-LNP system, it can induce maturation of dendritic cell cells, thereby enhancing antigen delivery and inducing subsequent immune responses. The antigens continue to be expressed *in vivo* after administration of the mRNA vaccine, stimulating the immune system to generate SEB-specific immune responses. The mSEB mRNA vaccine, administered at a lower dosage (10 μg), induced stronger immune responses compared to mSEB protein (30 μg administered with alum adjuvant). This highlights the potency and efficiency of the mRNA vaccine platform in eliciting immune responses against bacterial toxins like SEB.

After mRNA injection, it is taken up by both antigen-presenting cells (APCs) and non-APCs in the body. Within APCs, the mSEB protein is expressed intracellularly and processed by proteasomes, generating antigenic peptides. These peptides are then transported to the endoplasmic reticulum via TAP transporters, where they bind to MHC class I molecules (MHC-I). The peptide-MHC-I complexes are subsequently transported to the cell surface, where they induce a SEB- specific T-cell immune response (38). Additionally, the ability to confer long-term protection is a critical indicator for assessing vaccine efficacy. The three-dose vaccination regimen with the mSEB mRNA vaccine not only elevated SEB-specific antibody levels but also sustained these levels over an extended period (more than 100 days after the initial immunization). These suggest that the mRNA vaccine can provide durable and effective protection against SEB and potentially *S. aureus* infections. However, the specific processes and molecular mechanisms underlying the induction of immune responses—both humoral and cellular—following mRNA vaccine injection have not been thoroughly explored in this article. This area warrants further investigation in future research.

The data demonstrate that the neutralizing activity of the mRNA antibody against SEB exhibits dose dependence *in vivo*. anti-SEB mRNA antibodies exhibit a superior pharmacokinetic profile compared to the original monoclonal antibodies in protein format. Specifically, the serum concentration of the antibody after mRNA administration exceeded 40 μg/mL at a dose of 0.5 mg/kg, which is notably higher to previous reports (about 10-20 μg/mL at the same or higher doses) (32, 33, 39). In addition to the persistence of *in vivo* antibody expression, the sequence optimization and protective capacity of the original monoclonal antibodies against S. aureus also influence the efficacy of mRNA antibodies. The variable region sequences and the species origin of pathogen-specific antibodies play crucial roles in treatment of bacterial infections (40, 41). There are several technological challenges involved in screening and identifying functional antibody sequences (42, 43). Antibody sequences derived from clinical sources are more likely to exhibit high affinity for blocking bacteria in clinical settings. The original anti-SEB monoclonal antibody sequence used in our study was identified from clinical subjects, fully humanized, and well-suited for subsequent clinical applications. Regrettably, the SEB challenge model is acutely lethal to mice, causing acute mortality within 12 to 24 hours, which makes it unsuitable for evaluating the therapeutic efficacy of mRNA antibodies.

In conclusion, we developed mRNA-based vaccines and antibodies targeting SEB for both prophylactic and therapeutic purposes in varying *S. aureus* infection conditions. Our findings indicate that the mSEB mRNA vaccine shows promise as an effective prophylactic intervention against SEB and *S. aureus* infections. Additionally, mRNA antibodies offer an effective therapeutic option specifically targeting *S. aureus*. Our study highlights the potential of mRNA-based approaches in combating SEB and potentially broader infections caused by *S. aureus*, thereby addressing challenges associated with multidrug resistance in bacterial pathogens.

## Materials and methods

### Cell lines and bacterial strains

Human embryonic kidney (HEK) 293 cell line, RAW264.7 cell line, kuffer cell line were obtained from ATCC (Manassas, VA, USA). Cells were maintained in Dulbecco’s Modified Eagle’s Medium (DMEM, Gibco, USA) supplemented with 10% fetal bovine serum (Gibco, USA), penicillin (100 units/mL) and streptomycin (100 μg/mL) (complete medium) at 37°C in 5% CO_2_. All cell lines tested negative for mycoplasma contamination. *S. aureus* strain ST59 used in the study were routinely cultured in tryptic soy broth (TSB) and grown at 37°C in a shaking incubator at 200 rpm.

### Mice

6–8 weeks old specific pathogen-free (SPF) female BALB/c mice were purchased from the Beijing HFK Bioscience Co., Ltd (Beijing, China). All animal studies were approved by the Laboratory Animal Welfare and Ethics Committee of Third Military Medical University (AMUWEC20230059) and were performed in accordance with the institutional and national policies and guidelines for the use of laboratory animals. The mice were kept and vaccinated in SPF facilities, and provided with free access to sterile food and water. Animals were randomly divided into groups and conceded an adaption time of at least 7 days before the beginning of the experiments.

### Synthetic mRNA and LNP formulation

The coding sequence for mSEB protein or anti-SEB antibody (including heavy chain and light chain) were cloned into an IVT-mRNA production template plasmid carrying a T7 promoter, 5’ and 3’ UTR elements and Kozak consensus sequence. IVT-mRNA was produced using linearized IVT template plasmid and the MEGAScript T7 kit (HBP001505, Hzymes Biotech) and formulated with nucleoside-modified m1Ψ-5’-triphosphate instead of UTP. 5’-Capping and poly(A) of the IVT-mRNAs were performed co-transcriptionally using the trinucleotide cap1 analog (Cap1 capping system, HBP001513, Hzymes Biotech) and E.coli Poly(A) Polymerase (DD4111-01,Vazyme) The resulting capped mRNAs were purified by DNase I digestion, precipitated with LiCl and washed with 70% ethanol. mRNAs were stored at −80°C for further use.

The preparation processes of LNP are as follows: dissolving ionizable lipid (DLin-MC3-DMA), cholesterol, auxiliary lipid (DSPC), and polyethylene glycol in absolute ethanol at molar ratios of 50:38.5:10:1.5. The lipid mixture was combined with a 6.25 mM sodium acetate buffer (pH 5) containing mRNA (mRNA encoding the light and heavy chains at a 1:2 mass ratio or mRNA encoding mSEB) at a ratio of 1:3 (ethanol: aqueous) using a microfluidic device equipped with fishbone-type chips (Nexstar Shanghai Nano Technology Co., Ltd., Shanghai, China). Formulations were concentrated using Amicon ultra centrifugal filters (EMD Millipore), passed through a 0.22 μm filter, and stored at 4°C until use. All formulations were tested for particle size and RNA encapsulation. The LNP formulations was greater than 90% encapsulation with about 90 nm in size.

### Enzyme linked immunosorbent assay

SEB-specific antibodies in serum after administration of mSEB mRNA vaccine were measured by ELISA. Briefly, polystyrene microtiter 96-well plates were coated with SEB protein (3μg/mL in carbonate buffer, pH = 9.6) and incubated overnight at 4°C. After blocking with 1% bovine albumin (BSA) in PBS, 100 μL/well pre-diluted serum were added into the plates with 1 h incubation at 37 °C. After three-times washes with PBST (PBS with 0.05% Tween-20), plates were added with horseradish peroxidase (HRP) conjugated goat anti-mouse IgG (AS003, 1:10000, ABclonal) and incubated for 40 min at 37 °C. Plates were then washed three-times and added with peroxidase substrate (P0209, Beyotime), the reaction was terminated by stop solution (P0215, Beyotime) and the absorbances at 450 nm were read using a microplate reader (AID iSpot, Germany).

Evaluation of anti-SEB antibodies expression *in vitro* and *in vivo* after administration of anti-SEB mRNA antibody were performed by ELISA as described above except using the goat anti-human IgG (AB98624, 1:10000, Abcam) as the secondary antibody.

### Western blotting

We used western blot analysis to investigate whether the mRNA encoding mSEB can express intact protein *in vitro*. Briefly, HEK293T cells were transfected with mSEB mRNA and incubated for 24 hours. Subsequently, culture supernatant (Sup) and whole cell lysate (WCL) were collected and loaded onto gel electrophoresis. After electrophoresis, proteins were transferred to a PVDF membrane, which were then blocked and incubated with purified anti-SEB monoclonal antibody for 1 hour. Following this, a corresponding secondary antibody was applied, and the membrane was subjected to exposure detection.

To further investigated whether the mSEB mRNA vaccine is capable of inducing specific antibodies against wild-type SEB (wSEB) *in vivo*, purified wSEB and mSEB protein were loaded onto gel electrophoresis and transferred to a PVDF membrane. Serum was collected 35 days after the initial vaccination of mSEB mRNA vaccine as the primary antibody and subjected to western blot analysis following the aforementioned steps.

### Bone marrow derived dendritic cells maturation study

Bone marrow cells were isolated from the femurs of female BALB/c mice and cultured in RPMI 1640 complete medium (Gibco, USA) supplemented with 10% FBS, 1% penicillin/streptomycin, 10 ng/mL of Interleukin-4 (IL-4) and Granulocyte-Macrophage Colony Stimulating Factor (GM-CSF). The culture media was replaced with fresh media on day 2 and 5 to remove the non-adherent and loosely adherent cells. The remaining cells continued to culture for another 2 days. To examine the maturation of BMDCs *in vitro*, BMDCs (1 × 10^6^/mL) were co-cultured with mSEB protein and mSEB mRNA for 24 h, respectively. Subsequently, FITC anti-mouse CD11c (117305, Biolegend), PE anti-mouse CD40 (124609, Biolegend), PerCP/Cyanine5.5 anti-mouse CD80 (104721, Biolegend) and APC anti-mouse CD86 (17-0862-81, Invitrogen) were used to stain the cells in flow cytometry staining (FACS) buffer for 30 min at 4°C before being washed and analyzed by BD FACS Array software™ on a BD FACS Array flow cytometer (BD Biosciences, USA).

### Tissue processing and flow cytometry analysis

Single cell suspensions of splenic lymphocytes were prepared from resected spleens of mice. Splenic lymphocytes were collected by grinding spleen in PBS then passed through a 75 mm cell strainer, cell pellets were re-suspended in 5 mL of red blood cell lysis buffer (RT122-02, TIANGEN) for 5 min at RT for remove the red blood cells. PBS was added to wash the cells twice, then centrifuged at 1500 × g for 5 min, the cell pellets were eventually re-suspended in RPMI1640 media supplemented with 10% FBS and 1% penicillin/streptomycin.

For flow cytometric study, cells were first stained with the LIVE/DEAD fixable cell stains kit (423105, Biolegend) according to the manufacturer’s protocol. For surface markers, the cells were incubated with FITC anti-mouse CD4 (11-0041-82, Invitrogen), PerCP-Cy5.5 anti-mouse CD8a (45-0081-82, Invitrogen). For intracellular cytokine staining, cells were stimulated with the peptide pools of 15-mer overlapping peptides spanning the SEB protein (see **Additional File 1**: **Supplementary Table S1**) and protein transport inhibitor (BD GolgiStop 554724) for 6 h at 37°C, 5% CO_2_. Then the cells were incubated with APC anti-mouse IL-4 (504106, Biolegend) and PE anti-mouse IFN-γ (12-7311-82, Invitrogen) after processing with the Cytofix/Cytoperm Fixation/Permeabilization Kit (00-5523-00, Invitrogen) according to the manufacturer’s instructions. The antibodies were diluted 1:100 with stain buffer according to the manufacturer’s protocol. All the samples were measured on a BD FACS Array flow cytometer (BD Biosciences). Data are analyzed with FlowJo software V10.

### Enzyme linked immunospot assay

Cellular immune responses in mice were performed using mouse IFN-γ/IL-4 ELISPOT PLUS plates (3321-4APW-2/3311-4APW-2, MABTECH, Sweden). 96-well ELISPOT plates were pre-treated as the manufacturer’s instructions. 5 ×10^5^ mouse splenocytes were plated into each well and stimulated with the above-mentioned peptide pools at a final concentration of 20 μg/mL of total peptides per well. Additionally, PMA/Ionomycin were added as a positive control and RPMI 1640 media was used as a negative control. After incubation at 37 °C, 5% CO_2_ for 24 hours, the plates were washed with PBS and incubation with biotinylated anti-mouse IFN-γ or IL-4 antibody for 2 h at RT. Finally, TMB substrate solution were added to visualize the spots. Spots were scanned and quantified by an ImmunoSpot CTL reader. Spot-forming unit (SFU) per million cells was calculated by subtracting the negative control wells.

### Immunization with mSEB mRNA vaccine

BALB/c mice were immunized intramuscularly on day 0 and boosted with the same dose on day 14 and 28, respectively. Each mouse intramuscularly received 10 µg of mSEB mRNA vaccine, 30 µg of mSEB protein with alum adjuvant, phosphate buffered saline (PBS) was adopted as a negative control. Mice were sacrificed on day 35 for assessing cellular immune response. Mice were challenge with SEB or ST59 strains on day 35 and then to evaluate the preventive protection.

### Administration with anti-SEB mRNA antibody

BALB/c mice received 0.05, 0.25 or 0.5 mg/kg of anti-SEB mRNA antibody or purified anti-SEB antibody by i.v. administration at 24 hours before SEB challenge. For evaluating the therapeutic effect against *S. aureus*, mice were challenged with ST59 strains 1 hour after administration of anti-SEB mRNA antibody.

### SEB and S. aureus challenge

The toxin challenged of SEB in mice is an acute lethal model. Mice were intravenously administration of 10 μg SEB and 10 mg D-galactosamine and then monitored for survival rate for 72 hours. In S. aureus infection challenge model, mice were intravenous challenge of 1.0×10^8^ CFUs of S. aureus strain ST59. For organ bacterial burden analyses, the organs (heart, livers, spleens, lungs and kidney) were harvested from mice 24 hours post infection followed by homogenizing and plating of Luria agar plates for enumeration of bacterial CFU.

### Histopathology

Tissues from mice were fixed with perfusion fixative (formaldehyde) for 48 h, and embedded in paraffin according to standard histological assays. Then, tissues were stained with hematoxylin and eosin (H&E). Images were captured using Olympus BX51 microscope equipped with a DP72 camera.

### Statistics and analysis

Statistical analyses were performed using the GraphPad Prism 8.0 (GraphPad Software, USA). Data were expressed as mean ± standard deviation (SD). Comparisons between multiple conditions were analyzed using analysis of One-way ANOVA with Dunnett’s *post-hoc* test. Differences were considered statistically significant when *P* < 0.05. All of the experiments were successfully repeated at least twice with three or more biological replicates to ensure the reproducibility of the data.

## Data Availability

All datasets generated for this study are included in the article/**Supplementary Material**.

## References

[1] Lázár V Snitser O Barkan D Kishony R. Antibiotic combinations reduce Staphylococcus aureus clearance. Nature. (2022) 610:540–6. doi: 10.1038/s41586-022-05260-5 PMC9533972 36198788

[2] Rigaill J Gavid M Fayolle M Morgene MF Lelonge Y Grattard F. Staphylococcus aureus nasal colonization level and intracellular reservoir: a prospective cohort study. Eur J Clin Microbiol Infect Dis. (2023) 42:621–9. doi: 10.1007/s10096-023-04591-z 36964269

[3] Mohsen N Antimicrobial Resistance C. Global burden of bacterial antimicrobial resistance in 2019: a systematic analysis. Lancet. (2022) 399:629–55. doi: 10.1016/S0140-6736(21)02724-0 PMC8841637 35065702

[4] Mohsen N. Global mortality associated with 33 bacterial pathogens in 2019: a systematic analysis for the Global Burden of Disease Study 2019. Lancet. (2023) 400:2221–48. doi: 10.1016/s0140-6736(22)02185-7 PMC9763654 36423648

[5] Keim KC Horswill AR. Staphylococcus aureus. Trends Microbiol. (2023) 31:1300–1. doi: 10.1016/j.tim.2023.07.001 37487767

[6] Zhang A Wu H Chen X Chen Z Pan Y Qu W. Targeting and arginine-driven synergizing photodynamic therapy with nutritional immunotherapy nanosystems for combating MRSA biofilms. Sci Adv. (2023) 9:9116. doi: 10.1126/sciadv.adg9116 PMC10348676 37450586

[7] Kwiecinski JM Horswill AR. Staphylococcus aureus bloodstream infections: pathogenesis and regulatory mechanisms. Curr Opin Microbiol. (2020) 53:51–60. doi: 10.1016/j.mib.2020.02.005 32172183 PMC7244392

[8] Bae JS Da F Liu R He L Lv H Fisher EL. Contribution of staphylococcal enterotoxin B to Staphylococcus aureus systemic infection. J Infect Dis. (2021) 223:1766–75. doi: 10.1093/infdis/jiaa584 PMC8161638 32937658

[9] Turk S Yanpar H Baesmat AS Canli SD Cinar OE Malkan UY. Enterotoxins A and B produced by Staphylococcus aureus increase cell proliferation, invasion and cytarabine resistance in acute myeloid leukemia cell lines. Heliyon. (2023) 9:9743. doi: 10.1016/j.heliyon.2023.e19743 PMC10559070 37810000

[10] Choi JY Shin S Kim NY Son WS Kang TJ Song DH. A novel staphylococcal enterotoxin B subunit vaccine candidate elicits protective immune response in a mouse model. Toxicon. (2017) 131:68–77. doi: 10.1016/j.toxicon.2017.03.012 28359755

[11] Liu Y Song Z Ge S Zhang J Xu L Yang F. Determining the immunological characteristics of a novel human monoclonal antibody developed against staphylococcal enterotoxin B. Hum Vaccin Immunother. (2020) 16:1708–18. doi: 10.1080/21645515.2020.1744362 PMC7482898 32275466

[12] Kingwell K. Vaccines take a shot at antimicrobial resistance. Nat Rev Drug Discovery. (2018) 17:229–31. doi: 10.1038/nrd.2018.8 29520094

[13] Micoli F Bagnoli F Rappuoli R Serruto D. The role of vaccines in combatting antimicrobial resistance. Nat Rev Microbiol. (2021) 19:287–302. doi: 10.1038/s41579-020-00506-3 33542518 PMC7861009

[14] Jansen KU Knirsch C Anderson AS. The role of vaccines in preventing bacterial antimicrobial resistance. Nat Med. (2018) 24:10–9. doi: 10.1038/nm.4465 29315295

[15] La Guidara C Adamo R Sala C Micoli F. Vaccines and monoclonal antibodies as alternative strategies to antibiotics to fight antimicrobial resistance. Int J Mol Sci. (2024) 25:5487. doi: 10.3390/ijms25105487 38791526 PMC11122364

[16] Patel A DiGiandomenico A Keller AE Smith TRF Park DH Ramos S. An engineered bispecific DNA-encoded IgG antibody protects against Pseudomonas aeruginosa in a pneumonia challenge model. Nat Commun. (2017) 8:637. doi: 10.1038/s41467-017-00576-7 28935938 PMC5608701

[17] Deal CE Richards AF Yeung T Maron MJ Wang Z Lai Y-T. An mRNA-based platform for the delivery of pathogen-specific IgA into mucosal secretions. Cell Rep Med. (2023) 4:101253. doi: 10.1016/j.xcrm.2023.101253 37918405 PMC10694625

[18] Wilcox MH Gerding DN Poxton IR Kelly C Nathan R Birch T. Bezlotoxumab for prevention of recurrent Clostridium difficile Infection. N Engl J Med. (2017) 376:305–17. doi: 10.1056/NEJMoa1602615 28121498

[19] Johnson S Gerding DN. Bezlotoxumab. Clin Infect Dis. (2019) 68:699–704. doi: 10.1093/cid/ciy577 30020417

[20] Alameh M-G Semon A Bayard NU Pan Y-G Dwivedi G Knox J. A multivalent mRNA-LNP vaccine protects against Clostridioides difficile infection. Science. (2024) 386:69–75. doi: 10.1126/science.adn4955 39361752 PMC11719173

[21] Stadler CR Bahr-Mahmud H Celik L Hebich B Roth AS Roth RP. Elimination of large tumors in mice by mRNA-encoded bispecific antibodies. Nat Med. (2017) 23:815–7. doi: 10.1038/nm.4356 28604701

[22] August A Attarwala HZ Himansu S Kalidindi S Lu S Pajon R. A phase 1 trial of lipid-encapsulated mRNA encoding a monoclonal antibody with neutralizing activity against Chikungunya virus. Nat Med. (2021) 27:2224–33. doi: 10.1038/s41591-021-01573-6 PMC8674127 34887572

[23] Tai W Yang K Liu Y Li R Feng S Chai B. A lung-selective delivery of mRNA encoding broadly neutralizing antibody against SARS-CoV-2 infection. Nat Commun. (2023) 14:8042. doi: 10.1038/s41467-023-43798-8 38052844 PMC10697968

[24] Lin L Pei Y Li Z Luo D. Progress and challenges of mRNA vaccines. Imed. (2023) 1:e20220008. doi: 10.1002/INMD.20220008

[25] Pardi N Hogan MJ Porter FW Weissman D. mRNA vaccines a new era in vaccinology. Nat Rev Drug Discovery. (2018) 17:261–79. doi: 10.1038/nrd.2017.243 PMC5906799 29326426

[26] Rojas LA Sethna Z Soares KC Olcese C Pang N Patterson E. Personalized RNA neoantigen vaccines stimulate T cells in pancreatic cancer. Nature. (2023) 618:144–50. doi: 10.1038/s41586-023-06063-y PMC10171177 37165196

[27] Mayer RL Verbeke R Asselman C Aernout I Gul A Eggermont D. Immunopeptidomics-based design of mRNA vaccine formulations against Listeria monocytogenes. Nat Commun. (2022) 13:6075. doi: 10.1038/s41467-022-33721-y 36241641 PMC9562072

[28] Wang X Liu C Rcheulishvili N Papukashvili D Xie F Zhao J. Strong immune responses and protection of PcrV and OprF-I mRNA vaccine candidates against Pseudomonas aeruginosa. NPJ Vaccines. (2023) 8:76. doi: 10.1038/s41541-023-00672-4 37231060 PMC10209580

[29] Chen Z Meng C Mai J Liu Y Li H Shen H. An mRNA vaccine elicits STING-dependent antitumor immune responses. Acta Pharm Sin B. (2023) 13:1274–86. doi: 10.1016/j.apsb.2022.11.013 PMC10031366 36970194

[30] Thran M Mukherjee J Ponisch M Fiedler K Thess A Mui BL. mRNA mediates passive vaccination against infectious agents, toxins, and tumors. EMBO Mol Med. (2017) 9:1434–47. doi: 10.15252/emmm.201707678 PMC5623855 28794134

[31] Van Hoecke L Roose K. How mRNA therapeutics are entering the monoclonal antibody field. J Transl Med. (2019) 17:54. doi: 10.1186/s12967-019-1804-8 30795778 PMC6387507

[32] Deng YQ Zhang NN Zhang YF Zhong X Xu S Qiu HY. Lipid nanoparticle-encapsulated mRNA antibody provides long-term protection against SARS-CoV-2 in mice and hamsters. Cell Res. (2022) 32:375–82. doi: 10.1038/s41422-022-00630-0 PMC8866932 35210606

[33] Wu L Wang W Tian J Qi C Cai Z Yan W. Engineered mRNA-expressed bispecific antibody prevent intestinal cancer via lipid nanoparticle delivery. Bioengineered. (2021) 12:12383–93. doi: 10.1080/21655979.2021.2003666 PMC8810065 34895063

[34] Schlake T Thran M Fiedler K Heidenreich R Petsch B Fotin-Mleczek M. mRNA: A novel avenue to antibody therapy? Mol Ther. (2019) 27:773–84. doi: 10.1016/j.ymthe.2019.03.002 PMC6453519 30885573

[35] Zhu F-C Zeng H Li J-X Wang B Meng F-Y Yang F. Evaluation of a recombinant five-antigen Staphylococcus aureus vaccine: The randomized, single-centre phase 1a/1b clinical trials. Vaccine. (2022) 40:3216–27. doi: 10.1016/j.vaccine.2022.04.034 35473663

[36] Wang T Tang Y Tao Y Zhou H Ding D. Nucleic acid drug and delivery techniques for disease therapy: Present situation and future prospect. Imed. (2024) 2:e20230041. doi: 10.1002/INMD.20230041

[37] Tang W Liu J Ding B. Nucleic acid nanostructure for delivery of CRISPR/Cas9-based gene editing system. Imed. (2023) 1:e20220014. doi: 10.1002/INMD.20220014

[38] Pishesha N Harmand TJ Ploegh HL. A guide to antigen processing and presentation. Nat Rev Immuno. (2022) 22:751–64. doi: 10.1038/s41577-022-00707-2 35418563

[39] Kose N Fox JM Sapparapu G Bombardi R Tennekoon RN de Silva AD. A lipid-encapsulated mRNA encoding a potently neutralizing human monoclonal antibody protects against chikungunya infection. Sci Immunol. (2019) 4:eaaw6647. doi: 10.1126/sciimmunol.aaw6647 31101672 PMC6629435

[40] Li Z Li S Zhang G Peng W Chang Z Zhang X. An engineered bispecific human monoclonal antibody against SARS-CoV-2. Nat Immunol. (2022) 23:423–30. doi: 10.1038/s41590-022-01138-w 35228696

[41] Lloyd EC Gandhi TN Petty LA. Monoclonal antibodies for COVID-19. Jama. (2021) 325:1015–5. doi: 10.1001/jama.2021.1225 33544136

[42] Robinson WH. Sequencing the functional antibody repertoire-diagnostic and therapeutic discovery. Nat Rev Rheumatol. (2015) 11:171–82. doi: 10.1038/nrrheum.2014.220 PMC4382308 25536486

[43] Cheng J Liang T Xie Q Feng Z Meng L. A new era of antibody discovery: an in-depth review of AI-driven approaches. Drug Discovery Today. (2024) 29:103984. doi: 10.1016/j.drudis.2024.103984 38642702

