# Supplementary material for: mRNA-based platform for preventing and treating Staphylococcus aureus by targeted staphylococcal enterotoxin B
Source: Front Immunol. 2024 Nov 21;15:1490044. doi: 10.3389/fimmu.2024.1490044 (PMC11617584; doi:10.3389/fimmu.2024.1490044)
Supplement: Supplementary file 1 [file DataSheet1.docx]

Supplementary Material

**Supplementary Table**

**Supplementary Table1. Peptide pools of 15-mer overlapping peptides spanning the SEB protein**

| **NO** | **Sequence** | **NO** | **Sequence** |
| --- | --- | --- | --- |
| 1 | MESQPDPKPDELHKS | 16 | EHNGNQLDKYRSITV |
| 2 | PDELHKSSKFTGLME | 17 | KYRSITVRVFEDGKN |
| 3 | KFTGLMENMKVLYDD | 18 | VFEDGKNLLSFDVQT |
| 4 | MKVLYDDNHVSAINV | 19 | LSFDVQTNKKKVTAQ |
| 5 | HVSAINVKSIDQFRY | 20 | KKKVTAQELDYLTRH |
| 6 | SIDQFRYFDLIYSIK | 21 | LDYLTRHYLVKNKKL |
| 7 | DLIYSIKDTKLGNYD | 22 | LVKNKKLYEFNNSPY |
| 8 | TKLGNYDNVRVEFKN | 23 | EFNNSPYETGYIKFI |
| 9 | VRVEFKNKDLADKYK | 24 | TGYIKFIENENSFWY |
| 10 | DLADKYKDKYVDVFG | 25 | NENSFWYDMMPAPGD |
| 11 | KYVDVFGANAYYQCA | 26 | MMPAPGDKFDQSKYL |
| 12 | NAYYQCAFSKKTNDI | 27 | FDQSKYLMMYNDNKM |
| 13 | SKKTNDINSHQTDKR | 28 | MYNDNKMVDSKDVKI |
| 14 | SHQTDKRKTCMYGGV | 29 | SKDVKIEVYLTTKKK |
| 15 | TCMYGGVTEHNGNQL |  |  |

**Supplementary figures**


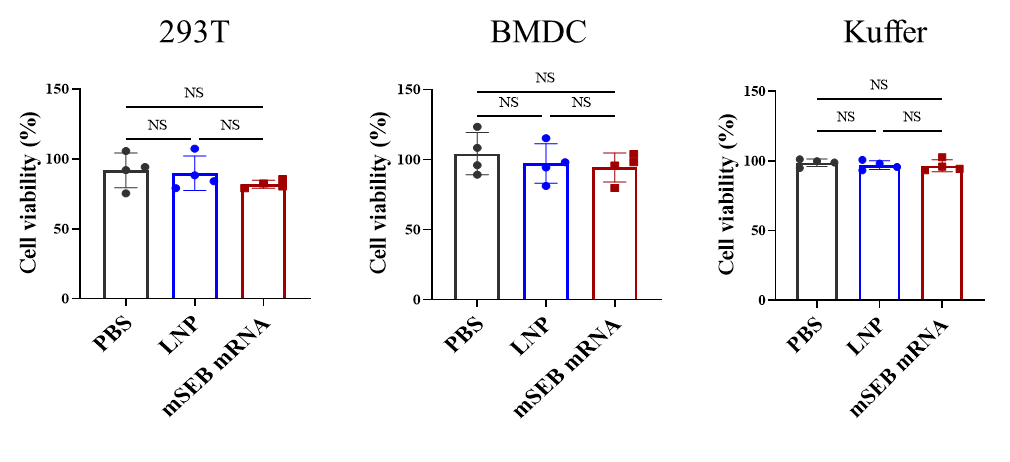


**Supplementary Figure 1.** Cytotoxicity of mSEB mRNA vaccine. CCK-8 cell viability assay of the mSEB mRNA vaccine, empty LNP and PBS after 24 hours incubation with different cell lines. Data represent mean ± SD (n = 4 independent experiments, NS represents no significant).


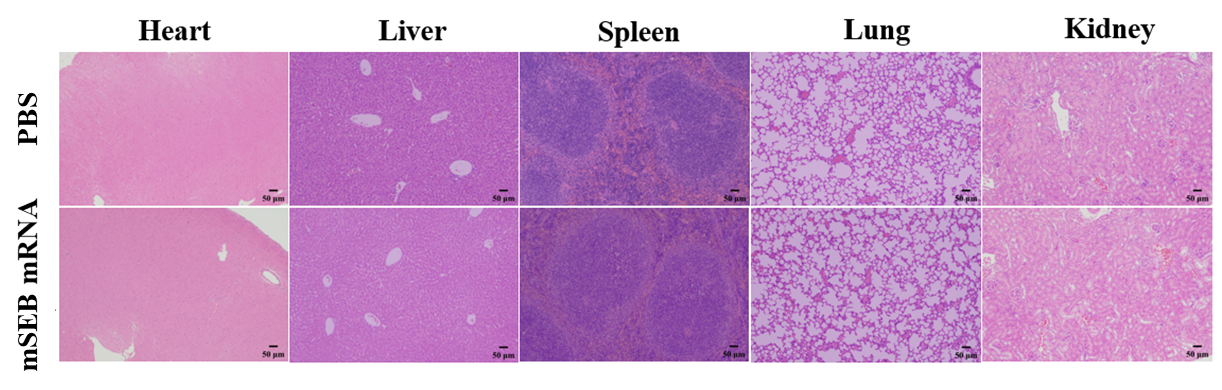


**Supplementary Figure 2.** Pathohistological analysis of tissue slides from mice in order to evaluate the biocompatibility of mSEB mRNA vaccine. Representative hematoxylin and eosin (H&E) staining of organs sections (including the heart, liver, spleen, lung and kidney) of mice collected 2 days after administration of mSEB mRNA. Control mice were treated with phosphate buffered saline (PBS). Scale bars: 50 μm.

**
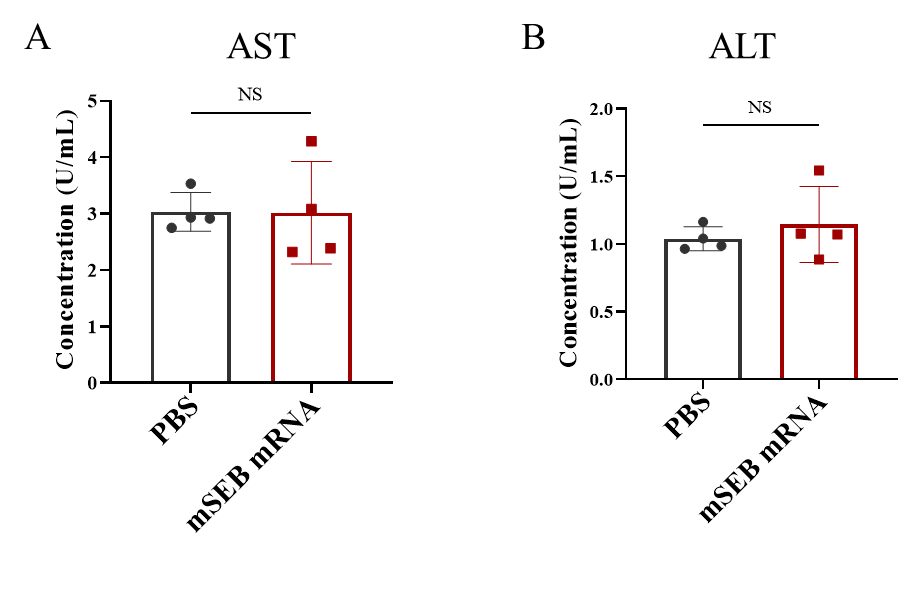
**

**Supplementary Figure 3.** Evaluation of liver function indices after immunization of mSEB mRNA vaccine. **(A)** aspartate aminotransferase (AST) and **(B)** alanine aminotransferase (ALT) was conducted 24 hours post-administration of the SEB mRNA vaccine. Data represent mean ± SD (n = 4 independent experiments, NS represents no significant).

**
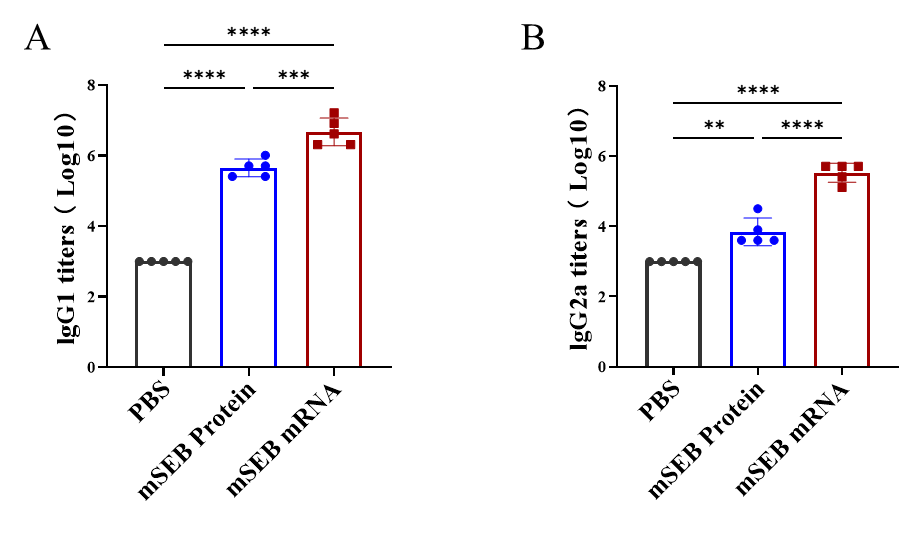
**

**Supplementary Figure 4.** The titers of **(A)** IgG1 and **(B)** IgG2a of PBS, mSEB protein and mSEB mRNA vaccine at 35 days after initial vaccination (n= 5 biologically independent samples). Data was shown as the mean ± SD. Statistical significance was calculated by one-way ANOVA with Dunnett’s multiple comparisons tests (***P*<0.01, ****P*<0.001,*****P*<0.0001).


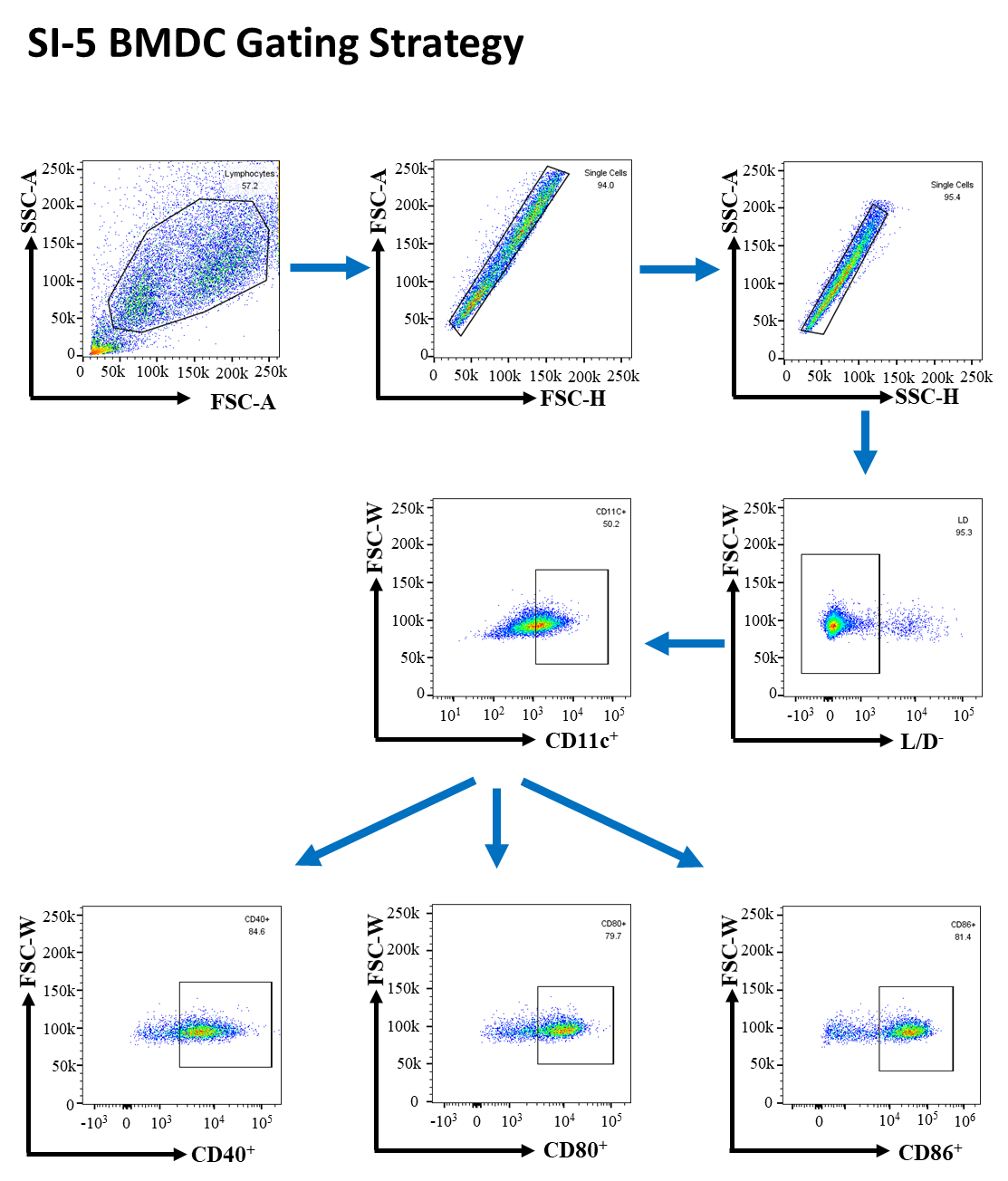


**Supplementary Figure 5.** Gating strategy used for the flow cytometry analysis as shown in FIGURE 2F-H.

**
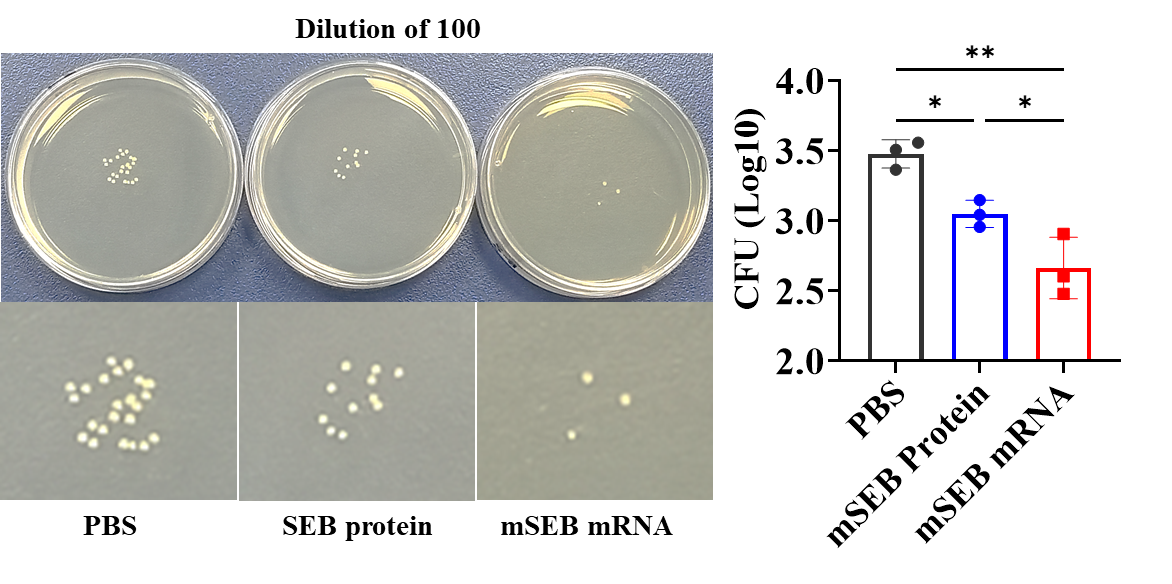
**

**Supplementary Figure 6.** Serum antibacterial activity. Serum samples collected from the PBS, mSEB protein (30 μg per mice with alum adjuvant) and mSEB mRNA vaccine groups were incubated with bacteria for 6 hours. Data represent mean ± SD (n= 3 biologically independent samples). One-way ANOVA with Dunnett’s post-hoc test was used to determine significance (*P< 0.05, **P< 0.01).


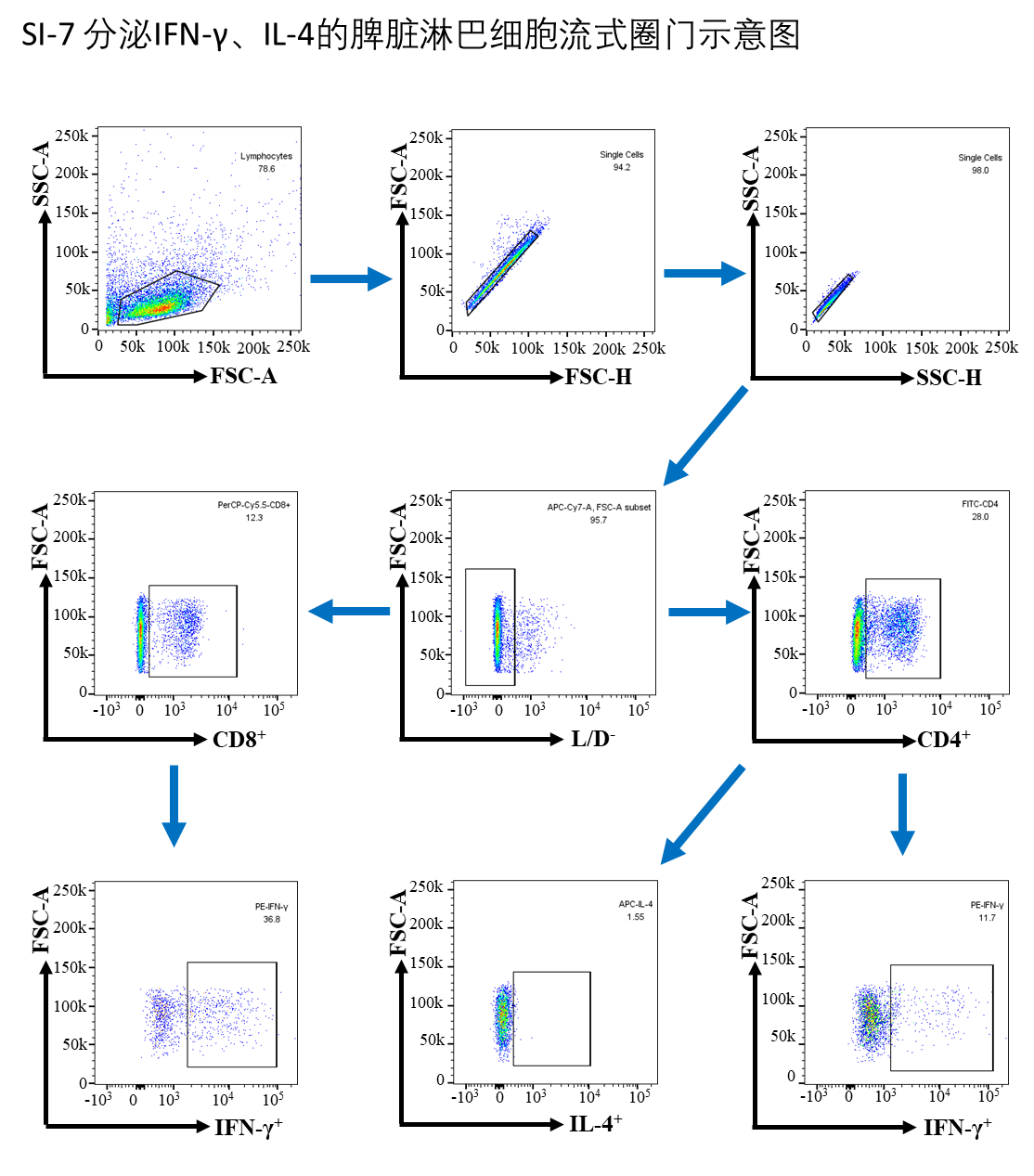


**Supplementary Figure 7.** Gating strategy used for the flow cytometry analysis as shown in FIGURE 3A-C.


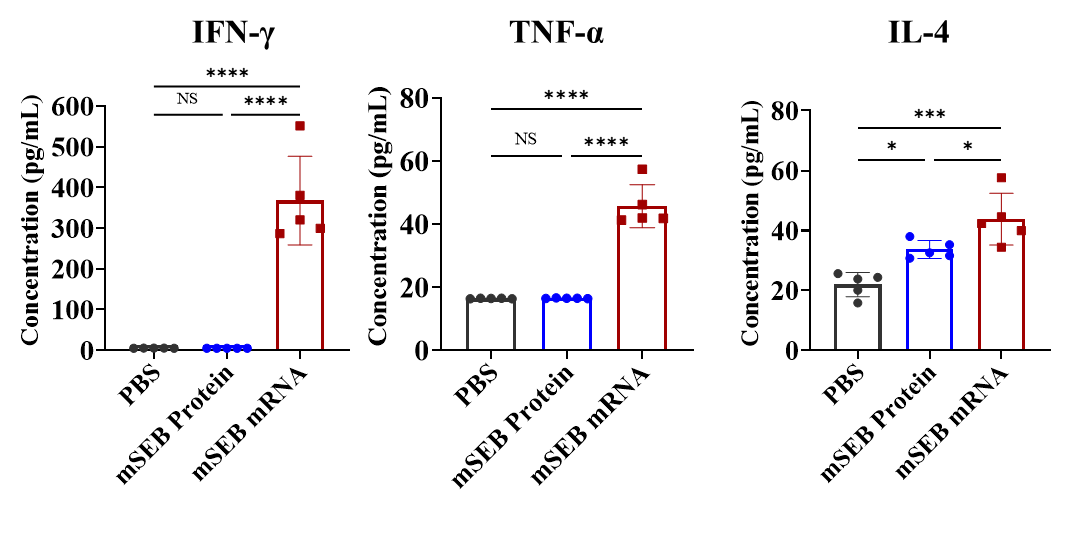


**Supplementary Figure 8.** Secretion of cytokine in the supernatants of splenic lymphocytes after re-stimulation with peptide pools. Data represent mean ± SD (n= 5 biologically independent samples). One-way ANOVA with Dunnett’s post-hoc test was used to determine significance (NS represents no significant ,*P< 0.05, ***P< 0.001, ****P< 0.0001).

**Supplementary Figure 9.** Expression levels of antibody in in Raw264.7, Expi293T and Expi293F cells at 24 hours after transfection with anti-SEB mRNA antibody by ELISA.

**
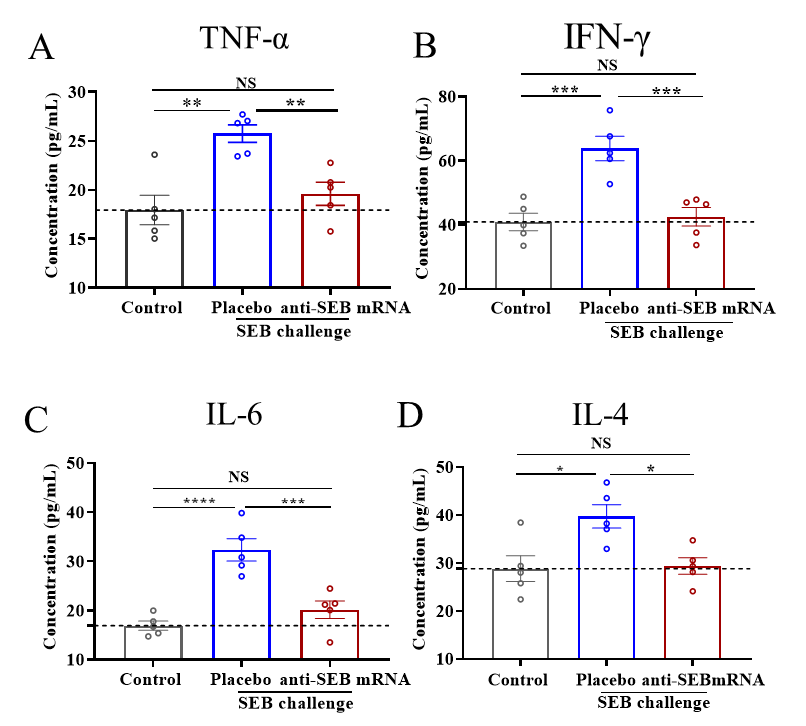
**

**Supplementary Figure 10.** Anti-SEB mRNA antibody inhibits super-antigen reactions induced by SEB. **(A)** The TNF-α, **(B)** IFN-γ, **(C)** IL-6 and **(D)** IL-4 expression in serum at 12h post SEB challenge. One-way ANOVA with Dunnett’s post-hoc test was used to determine significance (NS represents no significant, *P < 0.05, **P < 0.01, ***P < 0.001, ****P < 0.0001).

**
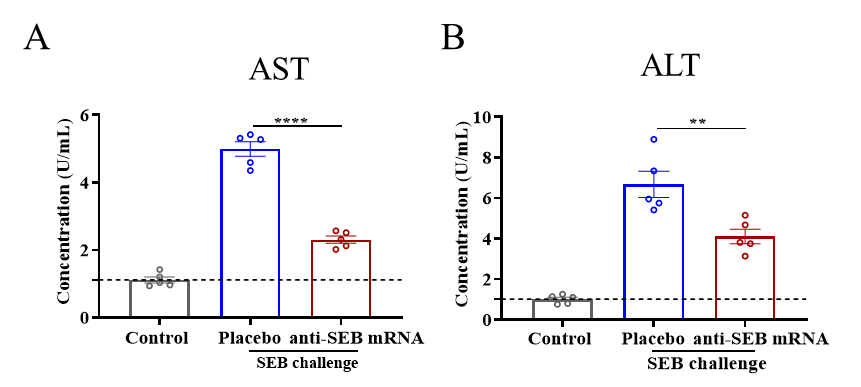
**

**Supplementary Figure 11.** Evaluation of liver function indices, including **(A)** aspartate aminotransferase (AST) and **(B)** alanine aminotransferase (ALT). The PBS and anti-SEB mRNA antibody groups were challenge with SEB and then serum were collected at 12 hours post challenge, serum from mice without challenge were used as control. One-way ANOVA with Dunnett’s post-hoc test was used to determine significance (**P < 0.01, ****P < 0.0001).
